# An Abnormal Fœtus

**Published:** 1902-04

**Authors:** J. H. Luff

**Affiliations:** Hudson, N. Y.


### REPORTS OF CASES.

### AN ABNORMAL FŒTUS.

By J. H. Luff, Ph.G., V.S.,
HUDSON, N. Y.

On March 18th I was called several miles in the country to deliver a calf from a Jersey heifer, the age of which was eighteen months. I arrived there about 10 o'clock at night, and found the patient in much pain and very uneasy.

I was told that she had been straining most of the day, and that a local "cow doctor" had been trying for three or four hours to deliver the calf.

As she was lying with the hindquarters in the gutter, I had her turned and the bedding arranged so as to have these parts the highest. She seemed to prefer the recumbent position, so I did not insist upon her getting up. I made an examination, and found pressing tightly into the passage what seemed to be a knee, but it was too large. What it was will be explained later.

The straining of the patient was so constant that I at once injected well into the uterus, with a small rubber hose three feet long and a bulb syringe, fluid extract lobelia, ℨij; glycerinum, ℥j; aqua fervens, O iv. I use the fluid extract because you can have the infusion instantly by mixing with the warm water, while the method of using the herb requires at least an hour, and this is valuable time to waste in a cold barn with arms bare and the outer clothing removed.

After ten or fifteen minutes I inserted the repeller and had an assistant push back the fœtus; I then searched for the feet, but could not reach them. The head was bent back on the side, and from the looseness of the teeth I knew the fœtus had been dead several days.

After several ineffectual attempts to pull the head straight I used the blunt hook in the intermaxillary space of the lower jaw; that brought the head up at once. But where were the front legs? After searching again and reaching as far as I could I concluded that we had an abnormality.

Without removing the hook (always hold what you get, or you may not get it soon again) I put a noose around the neck, and with some assistance delivered the calf. I then realized that what I

felt on the first examination was the inferior extremity of the scapula and point of shoulder.

Both of the front legs below the scapula were absent, although the skin at that point was smooth and entire, and the body was normal in every other way.

I thought a report of the case might be interesting in a pathological way, as I have never heard of a two-legged calf before.

